# The Importance of Metabolic Factors in Endometrial Cancer: Evaluating the Utility of the Triglyceride-to-Glycemia Index and Triglyceride-to-High-Density Lipoprotein Ratio As Biomarkers

**DOI:** 10.7759/cureus.62099

**Published:** 2024-06-10

**Authors:** Alina-Gabriela Marin, Radu Vladareanu, Aida Petca, Alexandru Filipescu

**Affiliations:** 1 Obstetrics and Gynaecology, Elias Emergency University Hospital, Bucharest, ROU; 2 Obstetrics and Gynaecology, Carol Davila University of Medicine and Pharmacy, Bucharest, ROU

**Keywords:** triglycerides-to-high density lipoprotein cholesterol ratio, triglycerides-fasting blood glucose index, lipid profile, endometrial hyperplasia, endometrial cancer

## Abstract

Introduction

Endometrial cancer (EC) is the most common gynecological malignancy in developed countries worldwide. Its incidence is rising, making it a significant public health concern. The relationship between lipids, hyperglycemia, and anthropometric risk factors in the development of EC has gained increasing attention in recent years. Understanding the role of dyslipidemia as a part of metabolic syndrome is crucial for developing effective prevention and treatment strategies for EC. We investigate the association between dyslipidemia, hyperglycemia, and EC. This study aims to elucidate the potential contribution of altered lipid profiles and chronic hyperglycemia to endometrial carcinogenesis. By analyzing patients with benign and malignant endometrial pathologies, we seek to identify novel biomarkers and unravel the underlying mechanisms by which these metabolic factors influence the risk of developing EC.

Material and methods

Our retrospective unicentric study included 390 patients (192 diagnosed with EC and 198 with endometrial hyperplasia), in which we compared the clinical and biochemical characteristics, with a particular focus on lipid profiles and glycemic indices sampled 24-48 hours before surgery. The data obtained from the medical records were analyzed using statistical methods to compare selected metabolic factors between EC and endometrial hyperplasia.

Results

Our analysis revealed statistically significant differences in metabolic health and lipid profiles between patients diagnosed with EC and those with endometrial hyperplasia. The EC group exhibits trends towards higher levels of triglycerides (TG) and glycated hemoglobin, alongside a higher BMI. Notably, high-density lipoprotein cholesterol levels were lower in the EC group.

Conclusion

Although the triglycerides-to-fasting blood glucose index and the triglycerides-to-high-density lipoprotein cholesterol ratio did not demonstrate sufficient discriminatory power for predicting myometrial invasion depth in this study, further exploration of cost-effective emerging biomarkers warrants investigation in future studies.

## Introduction

Endometrial cancer (EC) is a common gynecological malignancy, with its incidence increasing in peri- and postmenopausal women. Established risk factors for EC, including obesity, dysglycemia, and arterial hypertension, are well-studied within the framework of tumor metabolism [1]. This study bridges a knowledge gap by comprehensively analyzing the relationship between these two domains through the lens of lipid homeostasis.

Lipid homeostasis refers to the complex regulatory processes that govern the production, transport, and utilization of lipids in blood plasma, such as total cholesterol (TC), triglycerides (TG), high-density lipoprotein cholesterol (HDL-C), and low-density lipoprotein cholesterol (LDL-C). The lipid profile plays a very important role in maintaining overall health. Conventionally used to evaluate cardiovascular health, lipid profiles are becoming increasingly recognized for their emerging role in detecting endometrial abnormalities and influencing endometrial receptivity, a critical factor for successful embryo implantation. While dyslipidemia has been linked to various types of neoplasia, including cervical cancer, we aim to investigate its specific role in the development of EC [2,3].

Previously reported as a risk factor for EC and also considered an important component of metabolic syndrome, obesity frequently co-occurs with insulin resistance (IR). As IR progresses, target tissues become less responsive to insulin, leading to hyperinsulinemia. This hormonal dysregulation disrupts glucose homeostasis, potentially culminating in diabetes mellitus (DM) in severe cases. A significant correlation has been demonstrated between hyperinsulinemia and endometrial pathology, encompassing disordered proliferation, hyperplasia, and type I EC [4]. Emerging evidence suggests that insulin and its structurally similar counterpart, Insulin-like Growth Factor-1 (IGF-1), may play a role in malignant transformation and cancer progression through receptor activation [5]. Chronic hyperglycemia, a hallmark of uncontrolled DM, may further exacerbate these processes by increasing cellular sensitivity to IGF-1 and promoting a pro-angiogenic and anti-apoptotic microenvironment [6].

Moreover, research suggests that IR promotes cell proliferation and triggers oxidative stress and inflammatory processes, potentially fostering a tumor-permissive microenvironment. Furthermore, hyperinsulinemia may disrupt energy homeostasis by increasing cellular glucose uptake, potentially promoting cell proliferation and inhibiting apoptosis [7].

According to the initial definition of metabolic syndrome, IR was prioritized as the central element that translated into impaired fasting glucose (IFG), impaired glucose tolerance (IGT), or homeostatic high IR pattern assessment (HOMA-IR). The hyperinsulinemic-euglycemic clamp is the gold standard for IR diagnosis, which is impractical for routine clinical use because of its time-consuming, costly, and technically demanding nature [8]. The search for readily available and cost-effective alternatives for IR assessment in clinical practice is ongoing. The fasting triglyceride-glucose index (TyG) and TG/HDL-C have emerged as promising markers with established links to IR [9]. The TyG index's potential extends beyond IR assessment. Recent studies have demonstrated associations between elevated TyG levels and various cardiometabolic risk factors, including obesity and hypertriglyceridemia. Given the established link between obesity and IR, the TyG index is being explored as a potential tool for identifying individuals at heightened risk for obesity-associated malignancies [10,11].

This study aims to investigate potential differences in lipid profiles and hyperglycemia between patients with benign and malignant endometrial pathologies. By examining these two conditions side by side, we can identify any differences or similarities in the lipid profile and risk factors, which may help us better understand the underlying mechanisms and potential biomarkers associated with EC and endometrial hyperplasia (EH). This knowledge could ultimately lead to more effective prevention strategies and targeted treatments for these conditions. Moreover, investigating the lipid profile and risk factors in EC and EH can contribute to the growing body of literature on the association between lipid metabolism and gynecological cancers.

## Materials and methods

Study design

We designed this study as a retrospective cohort analysis to evaluate the association between hyperglycemia, lipid profile, and several modifiable risk factors including obesity and smoking in the development of EC.

Data source

We collected anonymized electronic health records from the Obstetrics and Gynecology clinic of Elias University Emergency Hospital in Bucharest, Romania. The data covered the period from January 1, 2015, to December 31, 2022.

Study population

In this study, we enrolled the first group of 192 women over 18 years old diagnosed with EC who underwent surgery based on their characteristics and clinical stage. Our second group included 198 women over 18 years old diagnosed with EH within the same timeframe as the EC group. These women underwent either an endometrial biopsy or a total abdominal hysterectomy with or without bilateral salpingo-oophorectomy (BSO), based on the patient's age and type of EH.

We excluded women with other gynecological cancers, those with missing or incomplete data for key study variables, and those with significant medical comorbidities.

Data collection

Following standardized protocols, anonymized data were extracted from electronic medical records. Extracted variables encompassed demographics (age, ethnicity, education level, employment status, marital status, parity), clinical and histopathological characteristics (BMI, histological type, tumor grade and stage, presence of lymph node involvement or distant metastases), and the following biochemical markers: fasting glucose, glycated hemoglobin (HbA1c), lipid profile (TC, LDL-C, HDL-C, TG), TyG index, and TG/HDL-c ratio.

Following established phlebotomy protocols, peripheral blood samples were collected in the fasting state from the patients 24-48 hours before surgical intervention. Standardized definitions and cut-off points were meticulously applied to ensure data integrity and comparability across all analytes.

To ensure clarity and consistency, the TyG index was calculated as the natural logarithm (Ln) of (fasting triglyceride level (mg/dL) × fasting plasma glucose (mg/dL)) divided by 2. The TG/HDL-c ratio represents the ratio of triglycerides (mg/dL) to high-density lipoprotein cholesterol (mg/dL).

Statistical analysis

We leveraged XLSTAT (version 2023.3.1.1416), a widely recognized statistical software package, to conduct the data analysis. Descriptive statistics were generated to comprehensively characterize the study population and evaluate the distribution of both clinical and biochemical markers. A significance level of α = 0.05 was predetermined to define statistically significant findings.

To assess group differences in clinical and biochemical characteristics, we employed appropriate statistical tests following an evaluation of continuous variable normality. For normally distributed data, the t-test for equality of means was used. Conversely, non-parametric tests were applied for variables with non-normal distributions. We adopted a data presentation strategy tailored to normality. Normally distributed data are presented as mean ± SD, depicting both central tendency and data dispersion within each group. This approach ensures a clear understanding of the distribution of these variables.

Additionally, within the EC group, receiver operating characteristic (ROC) curve analysis was undertaken to assess the diagnostic efficacy of the TyG index and TG/HDL-c ratio in differentiating the myometrial invasion grade.

Confidentiality and ethics

This study was approved by the Institutional Review Board of Elias University Emergency Hospital, Bucharest, Romania (approval number: 7172/26.08.2022). To ensure participant confidentiality, all data were anonymized before analysis.

## Results

This retrospective study enrolled a total of 390 patients, categorized into two groups: 192 diagnosed with EC and 198 diagnosed with EH. The mean age at diagnosis in the EC group was significantly higher at 62.42 years ± 10.62 compared to 59.16 years ± 10.74 in the EH group (p = 0.003). Patients with EC experienced menarche at a statistically younger age (11.11 years ± 1.10 vs. 13.74 years ± 1.20 in the EH group, p < 0.0001). Parity (mean: 1.52 children in EC vs. 1.85 children in EH) and number of abortions (mean: 2.05 in EC vs. 2.87 in EH) also revealed significant differences. The mean menopausal age (48.71 years in EC vs. 49.14 years in EH) did not demonstrate statistically significant differences between the groups. BMI analysis revealed a significant difference between the groups, with patients in the EC group displaying a higher mean BMI (36.58 ± 5.598) compared to those in the EH group (32.16 ± 5.98, p < 0.0001). Descriptive data are provided in Table 1.

**Table 1:** Baseline clinical characteristics: descriptive data for EC vs. EH. EC: Endometrial cancer; EH: Endometrial hyperplasia.

Variable	Analysed group	Observations	Obs. with missing data	Obs. without missing data	Minimum	Maximum	Mean	Std. deviation	Mean difference	95% CI	P-value
Age at diagnosis (years)	EC	192	0	192	33.00	93.00	62.42	10.62	3.26	1.13-5.38	0.003
	EH	198	0	198	38.00	87.00	59.16	10.74			
Menarche (years)	EC	192	0	192	8.00	14.00	11.11	1.10	-2.63	-2.86 to -2.40	<0.0001
	EH	198	0	198	9.00	18.00	13.74	1.20			
No. of abortions	EC	192	0	192	0.00	16.00	2.05	2.60	-0.82	-1.39 to -0.25	0.005
	EH	198	0	198	0.00	15.00	2.87	3.12			
No. of births	EC	192	0	192	0.00	5.00	1.52	1.14	-0.33	-0.58 to -0.08	0.010
	EH	198	0	198	0.00	9.00	1.85	1.34			
Menopausal age (years)	EC	192	0	192	30.00	59.00	48.71	5.31	-0.43	-1.52 to 0.65	0.434
	EH	198	41	157	28.00	57.00	49.14	4.88			
Weight (kg)	EC	192	0	192	60.00	167.00	94.72	14.45	9.10	6.04-12.16	<0.0001
	EH	198	0	198	54.00	170.00	85.62	16.23			
Height (m)	EC	192	0	192	1.47	1.78	1.61	0.05	-0.02	-0.03 to -0.01	<0.0001
	EH	198	0	198	1.49	1.80	1.63	0.06			
BMI (kg/m^2^)	EC	192	0	192	21.97	55.80	36.58	5.60	4.43	3.27-5.58	<0.0001
	EH	198	0	198	20.58	58.82	32.16	5.98			

Both Table 2 and Table 3 summarize the distribution of smoking habits among participants diagnosed with EC and EH. A cross-tabulation analysis reveals the distribution of cigarette consumption (packs) within the EC group. A substantial proportion (68.23%) were non-smokers. Among those with valid data (n=61, 31.77% of EC participants), the most frequent categories were smoking less than 10 cigarette packs (n=26, 13.54% of EC smokers) and 10-20 packs (n=27, 14.06% of EC smokers). Notably, a smaller proportion fell within the higher consumption categories (21-30 packs: n=5, 2.60%; >30 packs: n=3, 1.56%).

**Table 2:** Descriptive statistics: smoking habits and endometrial pathology. EC: Endometrial cancer; EH: Endometrial hyperplasia.

Smokers	EC	%	EH	%
Yes	61	31.77	109	55.05
No	131	68.23	76	38.38
Previous	0	0.00	13	6.57
Total	192	100.00	198	100.00

**Table 3:** Prevalence of EC and EH among smokers, stratified by number of cigarette packs smoked. EC: Endometrial cancer; EH: Endometrial hyperplasia.

Smokers	EC	% Group	% Total	EH	% Group	% Total
<10 packages	26	42.62	13.54	7	6.42	3.54
10-20 packages	27	44.26	14.06	60	55.05	30.30
21-30 packages	5	8.20	2.60	20	18.35	10.10
>30 packages	3	4.92	1.56	22	20.18	11.11
Total	61	100.00	31.77	109	100.00	55.05

When comparing smoking status across the EC and EH groups, a statistically significant difference is observed. A higher percentage of the EC group never smoked (68.2%) compared to the EH group (never smoker: 38.38%). Conversely, a higher proportion of the EH group were current smokers (55.05%) compared to the EC group (31.77%).

Table 4 synthesizes blood lipid profiles and glycemic indices in the EC and EH groups. Therefore, women with EC have slightly higher fasting glucose levels compared to those with EH. However, the high standard deviation in the EC group suggests a wide range of fasting glucose values within this group. We also observed that HbA1c, a marker of long-term blood sugar control, is slightly higher in the EC group (5.90%) compared to EH (5.43%).

**Table 4:** Comparison between blood lipid profiles and glycemic indices of EC and EH patients using descriptive statistics. Summary statistics (quantitative data). EC: Endometrial cancer; EH: Endometrial hyperplasia; HbA1c: Glycated haemoglobin; HDL-C: High-density lipoprotein cholesterol; LDL-C: Low-density lipoprotein cholesterol; TC: total cholesterol; TG: Triglycerides; TG/HDL-c: Triglycerides-to-high density lipoprotein cholesterol ratio; TyG index: Triglyceride-glucose index.

Variable	Analysed group	Observations	Obs. with missing data	Obs. without missing data	Minimum	Maximum	Mean	SD	Mean difference	95% CI	P-value
Fasting glucose (mg/dl)	EC	192	0	192	67.00	347.00	123.80	43.37	15.41	8.26-22.55	<0.0001
	EH	198	0	198	63.00	190.00	108.40	26.68			
HbA1c (%)	EC	192	0	192	4.00	11.40	5.90	1.31	0.47	0.24-0.70	<0.0001
	EH	198	0	198	3.80	9.40	5.43	0.96			
TC (mg/dl)	EC	192	0	192	83.00	320.00	221.69	47.28	6.55	-2.26-15.35	0.14
	EH	198	0	198	94.00	309.00	215.15	41.06			
LDL-C (mg/dl)	EC	192	0	192	26.00	255.20	141.68	41.38	1.17	-7.29-9.68	0.79
	EH	198	0	198	32.40	223.40	140.51	44.00			
HDL-C (mg/dl)	EC	192	0	192	22.00	75.00	45.47	9.56	-5.42	-7.33 to -3.51	<0.0001
	EH	198	0	198	29.00	69.00	50.90	9.58			
TG (mg/dl)	EC	192	0	192	72.00	364.00	172.67	58.30	53.99	44.19-63.79	<0.0001
	EH	198	0	198	56.00	322.00	118.68	38.40			
TyG index	EC	192	0	192	8.083	10.479	9.175	0.48	0.48	0.39-0.57	<0.0001
	EH	198	0	198	7.663	9.719	8.693	0.39			
TG/HDL-c ratio	EC	192	0	192	1.31	9.05	4.01	1.79	1.56	1.27-1.86	<0.0001
	EH	198	0	198	0.90	7.00	2.44	1.04			

EC patients exhibited a mean total cholesterol of 222.27 mg/dL, while EH patients had a mean of 215.15 mg/dL. LDL-C also showed a slightly upward trend in the EC group (141.68 mg/dL) compared to the EH group (140.51 mg/dL). Interestingly, HDL cholesterol was significantly higher in EH patients (50.90 mg/dL) compared to EC patients (45.47 mg/dL). Lastly, triglycerides were significantly elevated in the EC group (172.67 mg/dL) compared to the EH group (118.68 mg/dL).

**Figure 1:**
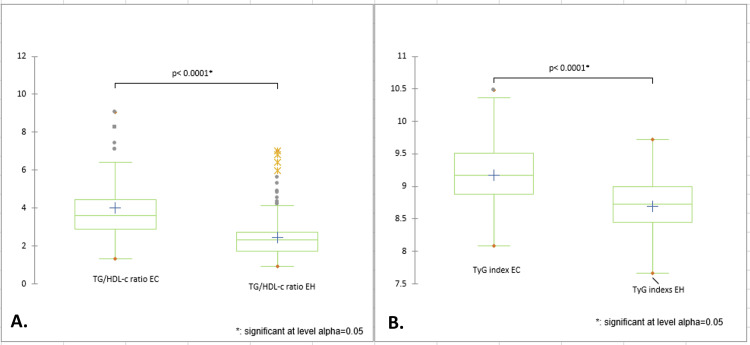
Differences between EC and EH groups: A. TG/HDL-c ratio. B. TyG index. This figure depicts significant differences (p < 0.0001) in lipid profiles between patients with EC and EH. The EC group exhibits a markedly elevated mean TyG index compared to the EH group. Additionally, the TG/HDL-c ratio is significantly higher in the EC group. EC: Endometrial cancer; EH: Endometrial hyperplasia; TG/HDL-c: Triglycerides-to-high density lipoprotein cholesterol ratio; TyG index: Triglyceride-glucose index.

Moreover, the EC group presented a statistically significant higher mean TyG index when compared to the EH group (mean difference 0.48, 95% CI 0.39-0.57, p < 0.0001). Likewise, the TG/HDL-c ratio also revealed a statistically significant difference (mean difference 1.56, p < 0.0001) between the groups. The 95% CI (1.27-1.86) suggests an elevated mean TG/HDL-c ratio in the EC group compared to the EH group (see Table 4 and Figure 1).

Moreover, we wanted to analyze the usefulness of these new markers in detecting myometrial invasion in the EC group. A ROC curve analysis was performed to evaluate the TyG index's ability to discriminate between less than 50% invasion and ≥50% invasion of the myometrium. The AUC of 0.586 indicates a poor to moderate discriminatory capacity.

**Figure 2:**
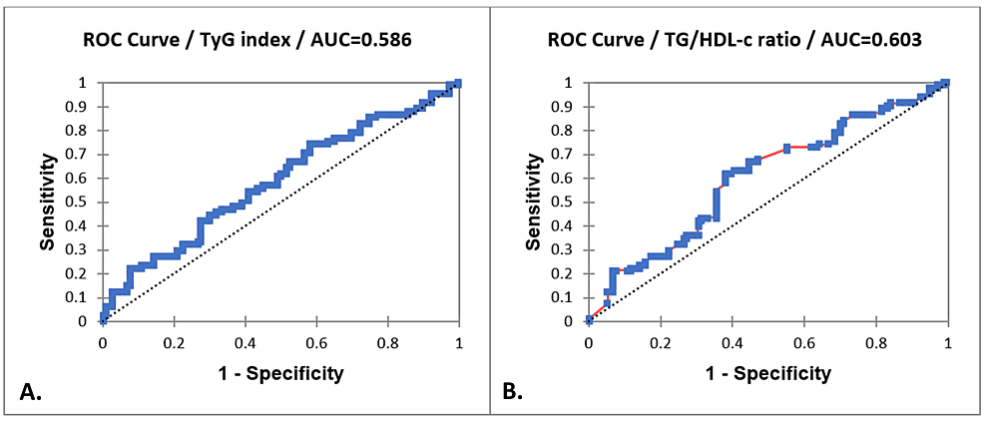
ROC curve analysis: A. TyG index; B. TG/HDL-C ratio in EC group and the association with myometrial invasion. ROC curve analysis assessed the ability of the TyG index and TG/HDL-c ratio to distinguish between shallow (<50%) and deep (≥50%) myometrial invasion in EC. Both markers yielded AUC values of 0.586 (TyG) and 0.603 (TG/HDL-c), indicating poor to moderate discriminatory power. EC: Endometrial cancer; TG/HDL-c: Triglycerides-to-high-density lipoprotein cholesterol ratio; TyG index: Triglyceride-glucose index.

Also, the ROC curve analysis for the TG/HDL-c ratio yielded an AUC of 0.603, suggesting a slightly better discriminatory ability compared to the TyG index, but still indicative of poor to moderate performance in differentiating between the groups (see Table 5 and Figure 2).

**Table 5:** The area under the curve for TyG index and TG/HDL-c ratio. TG/HDL-c: Triglycerides-to-high density lipoprotein cholesterol ratio; TyG index: Triglyceride-glucose index.

	AUC	Standard error	Lower bound (95%)	Upper bound (95%)
TyG index	0.586	0.042	0.505	0.668
TG/HDL-C ratio	0.603	0.042	0.521	0.685

## Discussion

Anthropometric factors

Age and BMI were identified as significant risk factors associated with EC. In our study, BMI, a widely used anthropometric measure, was significantly higher in the EC group compared to the EH group. This statistically significant difference indicates a greater prevalence of obesity within the EC population. Our study also showed that older age was correlated with an increased risk of developing EC.

A US study examined nearly 1,000 cases of EC and also observed a link between BMI and age at diagnosis with endometrioid adenocarcinoma. Women with a normal BMI were typically diagnosed at around 67 years old compared to women with a BMI exceeding 50, who were diagnosed at an average age of 56 years old. There was a strong association (p < 0.001) between higher BMI and younger age at diagnosis of EC [12].

A retrospective cohort study that included 4,164 Chinese women also observed a significant correlation between the BMI category and the prevalence of EC (p < 0.001). Multivariate analysis revealed that a BMI ≥ 25 kg/m² was independently associated with a 1.57-fold increased risk of EC (95% CI: 1.13-2.20, p = 0.008) compared to a BMI less than 25 kg/m² [13].

A study conducted in Switzerland and Italy included 458 women diagnosed with EC and 782 control participants. The analysis identified five distinct patterns of BMI throughout life. Participants in the 'Normal weight-stable' group served as the reference for comparison. Women whose BMI transitioned from 'Underweight' to 'Normal weight' experienced a significant reduction in EC risk, roughly 50% lower than the reference group. Those with a 'Normal weight increasing to overweight' trajectory had a slightly elevated risk (3%). However, the risk became considerably higher for women who remained 'Overweight-stable' throughout adulthood, with a 71% increase compared to the baseline. The highest risk was associated with the 'Overweight increasing to obese' trajectory, where the risk of EC more than doubled. Interestingly, the link between these BMI patterns and EC risk was even stronger for women who never used hormonal replacement therapy [14].

A Canadian meta-analysis investigated the association between excess BMI and oncological outcomes in EC survivors. Analysis of the 46 included studies revealed a significant association between higher BMI (obese, BMI ≥ 30 kg/m²) and increased risk of all-cause mortality and cancer recurrence in EC survivors. This association was observed for both type I and type II EC. Notably, no significant association was found between higher BMI and EC-specific mortality [15].

Glycaemic control

In our study, we noted slightly higher fasting glucose levels compared to those with EH. Also, HbA1c, a well-established indicator of long-term glycemic homeostasis, was marginally higher in the EC group (mean: 5.91%) compared to the EH group (mean: 5.06%). These observations suggest potentially suboptimal glycemic control in EC patients.

There is compelling evidence from a variety of study designs, including large cohort and case-control studies, to support a direct association between hyperglycemia and an increased incidence of EC. A Swedish meta-analysis examined the link between diabetes mellitus (DM) and EC through the evaluation of 16 studies. They observed a significant correlation between DM (in particular, type 2) and EC risk (RR 2.10, 95% CI 1.75-2.53) [16].

Another meta-analysis investigated the association between DM and EC incidence and mortality. It highlighted that DM was significantly associated with an increased risk of EC incidence (Summary Relative Risk [SRR], 1.81; 95% CI, 1.38-2.37). No significant association was observed between DM and EC mortality (SRR, 1.23; 95% CI, 0.80-1.90). However, moderate heterogeneity was present (I² = 58.2%), suggesting potential variation in mortality risk across studies [17].

Also, a meta-analysis comprehensively evaluated the association between DM and the risk of developing EC. After examining a total of 22 studies, encompassing both cohort and case-control designs, the analysis revealed a statistically significant positive association between DM and EC risk. Pooled data across all studies demonstrated a 72% increased risk of EC in diabetic women compared to non-diabetic controls (Relative Risk [RR] = 1.72, 95% CI, 1.48-2.01) [18].

Furthermore, a meta-analysis encompassing 31 studies (over 55,000 women diagnosed with EC) highlighted a statistically significant association between DM and poorer survival outcomes. Therefore, women with DM exhibited a 15% increased hazard ratio (HR) for EC-specific mortality compared to their counterparts without DM (HR 1.15, 95% CI 1.00-1.32). Patients diagnosed with both DM and EC demonstrated a higher risk of disease recurrence or progression (HR 1.23, 95% CI 1.02-1.47). Also, the presence of DM was associated with a 42% increased HR for all-cause mortality (HR 1.42, 95% CI 1.31-1.54) [19].

A Turkish retrospective study investigated the association between pre-operative HbA1c levels and the risk of concurrent EC in women diagnosed with Endometrial Intraepithelial Neoplasia (EIN). They enrolled 113 diabetic women: 29 diagnosed with benign abnormalities, 34 with EIN, and 50 with EC. Statistically significant differences (p < 0.001) were observed in pre-operative HbA1c levels between the groups. The highest mean HbA1c was found in the EC group (6.65%), followed by EIN (6.01%) and the benign group (5.41%). This trend persisted after adjusting for age and duration of menopause (p < 0.001). ROC analysis identified an HbA1c cut-off value of ≥6.05% for predicting concurrent EC in EIN patients, with a sensitivity of 60% and a specificity of 70% (p < 0.001) [20].

Moreover, a recent cohort study observed a correlation between HbA1c levels and the invasiveness of EC. HbA1c levels displayed a significant positive association with both EC stage and grade (p < 0.001 for both). The average HbA1c was notably higher in the advanced-stage group (10.85 ± 1.15) compared to the early-stage group (7.88 ± 1.35). Similarly, HbA1c levels were elevated in patients with high-grade tumors compared to those with low-grade tumors. Notably, no statistically significant difference in HbA1c levels was observed across different EC subtypes (p = 0.757) [21].

Lipid profile

The comparative analysis revealed significant differences in the lipid profiles of participants with EC and EH. Specifically, we found that individuals with EC exhibited higher levels of TG compared to those with EH. In contrast, HDL-C levels were lower in the EC group. These findings suggest a distinct lipid profile associated with EC compared to EH.

HUNT-II study demonstrates a positive and dose-dependent association between elevated serum TG levels and EC risk. Their findings revealed a statistically significant positive association, with women in the highest quartile of TG exhibiting a more than two-fold elevated risk of EC compared to those in the lowest quartile (HR = 2.34, 95% CI: 1.04-5.28) [22]. Furthermore, the Swedish AMORIS study investigated the potential association between serum TG levels and the risk of developing EC. They observed a statistically significant positive trend between increasing TG quartiles and EC risk (p-value for trend <0.001) [23].

UK study contributes to the growing body of evidence regarding the potential association between dyslipidemia and the risk of obesity-related malignancies. They employed well-established clinical cut-off points to define abnormal levels of key lipid parameters: TC ≥ 6.50 mmol/L, TG ≥ 1.71 mmol/L, HDL-C ≤ 1.03 mmol/L, and apolipoprotein A-I (ApoA-I ≤ 1.05 mmol/L). This analysis revealed a consistent pattern, with elevated levels of TC and TG emerging as significant risk factors for these cancers. Conversely, low levels of HDL and ApoA-I were also linked to a heightened risk [24].

A Japanese study, that included 412 women with EC, investigated the association between lipid profiles and EC risk. Interestingly, the association between hypertriglyceridemia and EC appeared to be particularly pronounced in the premenopausal BSO group. In this subgroup, women with elevated TG demonstrated a 2.26-fold increased OR of developing EC compared to those with normal TG levels. Analysis revealed a significant elevation in the LDL-C/HDL-C ratio (indicative of atherogenic dyslipidemia) within the premenopausal BSO group with EC compared to controls (p = 0.012). Additionally, the OR for EC in this group was 1.72 for a higher LDL-C/HDL-C ratio. These findings suggest a possible role for altered lipoprotein metabolism, particularly in premenopausal women following BSO, in the development of EC [25].

Increasing evidence suggests a potential protective role of HDL-C in cancer development. Epidemiological studies have documented an inverse association between HDL-C levels and certain malignancies, including breast, endometrial, lung, colorectal and liver cancer [26,27].

Recent cohort studies have also provided promising data demonstrating a correlation between low HDL-C levels and an increased risk of developing various individual cancers. Furthermore, a meta-analysis, a large-scale analysis of several studies, demonstrated a significant inverse correlation between HDL-C levels and overall cancer risk. However, this association is not uniformly observed, with some studies failing to establish a statistically significant association for breast and colorectal cancers [28].

New emerging biomarkers

In our analysis, the EC group exhibited a statistically significant higher mean TyG index when compared to the control group.

An Austrian study of 16,052 people investigated the role of the TyG index as a marker for IR and risk for the development of obesity-related cancer. The analysis showed a statistically significant positive association between the TyG index and the risk of developing cancer of kidney (HR: 1.13, 95% CI: 1.07-1.20); liver (HR: 1.13, 95% CI: 1.04-1.23), pancreas (HR: 1.12, 95% CI: 1.06-1.19), colon (HR: 1.07, 95% CI: 1.03-1.10), rectum (HR: 1.09, 95% CI: 1.04-1.14). In addition, the TyG index was found to be a significant mediator of the effect of BMI on the risk of developing pancreatic (42%), rectal (34%) and colon (20%) cancers. A moderate mediating effect was observed for kidney (15%) and liver (11%) cancers. In particular, the TyG index showed little or no mediating effect on the risk of breast, endometrial and ovarian cancers postmenopause. Limitations of the study included incomplete data on specific covariates, measurement heterogeneity through variations in TG and glucose measurement protocols between participating cohorts, and limited characterization of obesity subtypes [11].

Chinese meta-analysis of 6 observational studies encompassing a total of 992,292 participants was conducted to evaluate the association between the TyG index and cancer risk. The analysis revealed a statistically significant positive association between a higher TyG index and increased cancer risk (total effect size = 1.14, 95% CI 1.08, 1.20, P < 0.001) [7].

Another Chinese study investigated the association between the TyG index and the risk of EC. Patients diagnosed with EC or atypical endometrial hyperplasia (AEH) had significantly higher levels of the TyG index compared to a control group with normal endometrium (P < 0.001). Furthermore, a statistically significant positive trend (P < 0.001) was observed with a progressively increasing incidence of both EC and AEH along the TyG index tertiles, suggesting a potential dose-dependent relationship. They also confirmed that the TyG index remained an independent risk factor for both AEH (OR 2.54, 95% CI 1.33-4.85, P = 0.005) and EC (OR 2.65, 95% CI 1.60-4.41, P < 0.001). In addition, a high TyG index showed a positive correlation with a more advanced pathological stage (OR 2.14, 95% CI 1.32-3.47, P = 0.002) and poorer tumour differentiation (OR 2.53, 95% CI 1.36-4.72, P = 0.004) [29].

In our analysis, the TG/HDL-c ratio revealed a statistically significant difference between the compared groups.

A retrospective study investigated the association between pre-treatment serum lipid profile and TG/HDL-c ratio in 167 postmenopausal women with EC and 464 non-cancer controls. It evidenced a significantly elevated TG/HDL-c ratio in the EC group compared to controls (P < 0.05), regardless of pre-existing diabetes or overweight/obese status. Furthermore, a positive correlation was observed between the pre-treatment TG/HDL-c ratio and the advanced tumor stage (adjusted r = 0.176, P = 0.027). Multivariate logistic regression analysis further identified a pre-treatment TG/HDL-c ratio ≥ 1.52 as an independent predictor of EC (OR = 4.123; P < 0.001). In addition, patients with type I EC had a significantly higher TG/HDL-c ratio compared to those with type II EC [1].

Although the current investigation contributes to our understanding of the influence of metabolic factors on the development and invasiveness of EC, it is essential to acknowledge inherent limitations. Our study is a retrospective, single-center study. For this reason, the potential influence of confounding variables such as socioeconomic status, dietary habits, and physical activity levels could not be fully explored. These factors may independently influence both smoking behavior, and endometrial health. Also, a limitation is determined by the poor to moderate discriminatory power of the biomarkers used (TyG index and TG/HDL-c ratio) for the depth of myometrial invasion. Further investigation of emerging and cost-effective biomarkers for EC is warranted given the limited existing data. Future research efforts could explore their utility in stratifying between type I and type II ECs, as well as evaluating expression patterns of these biomarkers in patients diagnosed with simple and complex hyperplasia with atypia. It may be advantageous to develop multi-marker panels for enhanced diagnostic accuracy and risk stratification. Combining the discriminatory power of several biomarkers could provide a more robust and reliable classification system. Therefore, future prospective, multicenter studies with larger and more diverse populations are needed to confirm these findings and elucidate the underlying biological mechanisms. This exploration holds promise for improving diagnosis, risk stratification, and ultimately patient outcomes.

## Conclusions

Our analysis revealed significant disparities in metabolic health and lipid profiles between patients diagnosed with EC and EH. The EC group showed a trend toward a more negative metabolic profile, characterized by statistically elevated TG and HbA1c levels, along with a higher mean BMI. Also, HDL-C levels were reduced in the EC group. While the TyG index and TG/HDL-c ratio did not demonstrate robust discriminatory power for myometrial lesion invasiveness in this study, the exploration of alternative markers remains an important avenue for future research efforts. In conclusion, this study highlights the critical role of incorporating a comprehensive analysis of the lipid profile and established risk factors into the clinical assessment and management of both EC and EH. Identifying specific lipid biomarkers and risk factors associated with these gynecological pathologies can significantly contribute to developing early detection strategies and targeted interventions for high-risk individuals.
